# Inflammation, Thrombosis, and Destruction: The Three-Headed Cerberus of Trauma- and SARS-CoV-2-Induced ARDS

**DOI:** 10.3389/fimmu.2020.584514

**Published:** 2020-09-25

**Authors:** Ludmila Lupu, Annette Palmer, Markus Huber-Lang

**Affiliations:** Institute of Clinical and Experimental Trauma-Immunology, University Hospital Ulm, Ulm, Germany

**Keywords:** trauma, severe acute respiratory syndrome coronavirus 2, acute respiratory distress syndrome, lung, pathophysiology

## Abstract

Physical trauma can be considered an unrecognized “pandemic” because it can occur anywhere and affect anyone and represents a global burden. Following severe tissue trauma, patients frequently develop acute lung injury (ALI) and/or acute respiratory distress syndrome (ARDS) despite modern surgical and intensive care concepts. The underlying complex pathophysiology of life-threatening ALI/ARDS has been intensively studied in experimental and clinical settings. However, currently, the coronavirus family has become the focus of ALI/ARDS research because it represents an emerging global public health threat. The clinical presentation of the infection is highly heterogeneous, varying from a lack of symptoms to multiple organ dysfunction and mortality. In a particular subset of patients, the primary infection progresses rapidly to ALI and ARDS. The pathophysiological mechanisms triggering and driving severe acute respiratory syndrome coronavirus 2 (SARS-CoV-2)-induced ALI/ARDS are still poorly understood. Although it is also generally unknown whether insights from trauma-induced ARDS may be readily translated to SARS-CoV-2-associated ARDS, it was still recommended to treat coronavirus-positive patients with ALI/ARDS with standard protocols for ALI/ARDS. However, this strategy was questioned by clinical scientists, because it was documented that some severely hypoxic SARS-CoV-2-infected patients exhibited a normal respiratory system compliance, a phenomenon rarely observed in ARDS patients with another underlying etiology. Therefore, coronavirus-induced ARDS was defined as a specific ARDS phenotype, which accordingly requires an adjusted therapeutic approach. These suggestions reflect previous attempts of classifying ARDS into different phenotypes that might overall facilitate ARDS diagnosis and treatment. Based on the clinical data from ARDS patients, two major phenotypes have been proposed: hyper- and hypo-inflammatory. Here, we provide a comparative review of the pathophysiological pathway of trauma-/hemorrhagic shock-induced ARDS and coronavirus-induced ARDS, with an emphasis on the crucial key points in the pathogenesis of both these ARDS forms. Therefore, the manifold available data on trauma-/hemorrhagic shock-induced ARDS may help to better understand coronavirus-induced ARDS.

## Introduction

### Trauma as a Major Individual and Global Threat

Trauma is the leading cause of mortality among young people (aged 1–44 years) (1). It was estimated that the annual global mortality rate from trauma is more than five million (2). Apart from claiming lives, it can cause various disabilities of different degrees with lifelong consequences. Because trauma requires immediate hospitalization, prevention, and treatment of physical and psychological sequela, it is also associated with a high financial burden on society. Most of the trauma-related deaths are caused by road traffic accidents, followed by suicide, homicide, falls, fire-related burns, poisoning, and wars (2). According to the WHO, the incidence of road traffic injuries (the main cause of morbidity and mortality among young people) and falls (the main cause of morbidity and mortality among older individuals) will increase by 2030 (2). This data supports the concept that trauma, as an “unrecognized pandemic,” was in the past, is at present, and will remain in the near future a major burden for the individual as well as for the global population. Any severe tissue trauma can cause acute lung injury (ALI) and acute respiratory distress syndrome (ARDS). Post-traumatic ARDS is also particularly linked to older patients, a higher median Injury Severity Score (ISS), blunt mechanisms, and chest injury (3, 4). Furthermore, severe sepsis as a major consequence of accidental or surgical tissue trauma represents the most important driver for ARDS induced by pulmonary or nonpulmonary complications (3).

Different etiologies of ARDS, including trauma and sepsis, may induce a differential organ and immune response, based on the recent omics data. Overall a large overlap of similarly expressed genes and generated proteins has been detected when the immune response is transgressive, proposed as an “emergency response” (5). However, to date, it has not been addressed whether the organ and immune responses of intensive care unit (ICU) patients with trauma-induced ARDS share similar innate immune features as ICU patients with coronavirus-induced ARDS, which is the comparative focus of this review.

### Coronavirus as a Major Individual and Global Threat

The coronavirus family has been responsible for three large-scale pandemics in the last two decades. In 2002, the WHO declared the first pandemic caused by a coronavirus, namely by the severe acute respiratory syndrome coronavirus 2 (SARS-CoV-2), and this was the first pandemic of the 21^st^ century. The outbreak of SARS occurred in China, and from November 2002 until July 2003, there were 8096 cases and 774 deaths reported globally to the disease (6), with a case fatality ratio (CFR) of 9.6% (7). Ten years later, in 2012, the Middle East respiratory syndrome (MERS) coronavirus gave rise to the second coronavirus pandemic. Since 2012, ca. 2220 laboratory-confirmed cases and 790 deaths because of this disease have been reported (8), with a CFR of 34.3% (9). It is worth mentioning that while SARS-CoV pandemic was declared to be over, with no more infections being detected since 2003, the MERS-CoV continues to spread with new cases being reported every year (6). The third pandemic started in December 2019, being caused by SARS-CoV-2, leading to coronavirus disease 2019 (COVID-19), which in the meantime resulted in 10,533,779 laboratory-confirmed cases globally, including 512,842 deaths by the 2nd of July 2020 (10). The COVID-19 CFR was proved to be best stratified by age, where the CFR in the age group <60 years was estimated to be 1.4%, while in the age group ≥60 years—4.5% (11). Being considered a global threat, SARS, MERS, and COVID-19 are included in the priority diseases list of WHO for research and development (12).

ARDS, as an early consequence after primary infection of coronavirus, could be detected as the most common complication, particularly in older and in critically ill patients (13). Coronavirus-induced ARDS was defined as a specific ARDS phenotype because of its dissociation between the relatively well-preserved lung mechanics and the severity of hypoxemia. Therefore, an adjusted therapeutic approach in response to the underlying triggering mechanism appears to be required (14).

### Acute Respiratory Distress Syndrome

Irrespective of the underlying cause, whether traumatic or infectious (or both), ARDS is a life-threatening condition, characterized by pulmonary infiltrates and impaired oxygenation. The diagnosis of ARDS is currently based on the Berlin definition: acute onset (within 7 days of insult) or worsening respiratory symptoms, noncardiogenic pulmonary edema, radiologically diffuse bilateral infiltrates, and hypoxic respiratory failure with a Horovitz index ≤300 mmHg. The Horovitz index, being defined as the ratio of the arterial partial pressure of oxygen (PaO_2_) to the fraction of inspired oxygen (FiO_2_), is used to classify the ARDS severity degrees as follows: mild (PaO_2_/FiO_2_ = 201–300 mmHg), moderate (PaO_2_/FiO_2_ = 101–200 mmHg), and severe (PaO_2_/FiO_2_ ≤ 100 mmHg), when the positive end-expiratory pressure (PEEP) is ≥5 cm H_2_O (15).

Population-based, the incidence of ARDS varies greatly in high- and middle-income countries from 10.1 to 86.2 per 100,000 person-years (16). Reliable epidemiological data about ARDS in low-income countries is unavailable. The Berlin criteria have a low applicability in low-income countries, mainly because of the shortage of ICUs and ICU beds and a lack of imaging equipment and mechanical ventilation devices (16). A prospective cohort study (LUNG SAFE), conducted in 2014, collected and analyzed data from 459 ICUs from 50 countries across five continents. Based on this study, the ARDS prevalence of total ICU admissions was 10.4%. The reported mortality rate was 34.9, 40.3, and 46.1% for patients with mild, moderate, and severe ARDS, respectively (17). This observational study also showed that despite the simplicity of the Berlin criteria, ARDS remained underdiagnosed many times by the clinicians, with only 60.2% of all patients with ARDS being recognized. Moreover, regardless of the advance in supportive care, ARDS mortality remains high (17). The studies conducted before the implementation of the Berlin criteria similarly showed an ALI/ARDS mortality rate >40% (3, 18).

### Etiology

A plethora of pulmonary as well as extrapulmonary risk factors has been reported to play a role in ARDS induction. Blunt chest trauma, pneumonia, aspiration, inhalation injury, and ventilator-induced lung injury (VILI) are among the primary or pulmonary causes of ARDS. Secondary conditions playing a significant role in triggering ARDS are, for example, hemorrhagic shock (HS), systemic inflammatory response syndrome (SIRS), sepsis, acute pancreatitis, burns, and transfusion-associated acute lung injury (TRALI) (19). Several large cohort studies reported pneumonia in 35–50% of ARDS patients as being the most common risk factor for ARDS, followed by sepsis (30%), aspiration (10%), and trauma (10%) (17, 20–22).

### Pathogenesis

The primary role of the innate immunity is as an effective host defense. However, there is a fairly thin line between protective action and an insufficient or injurious immune response. In the case of ARDS, the host immune system in general exceeds its protective role and thereby may become detrimental. A hyperactivation of the host immune system might be followed by its exhaustion and subsequent insufficiency, which could be mirrored by clinical data of patients with the so-called hyper- and hypo-inflammatory ARDS phenotypes (23).

Viral or bacterial infection, acid exposure, hypoxia, and mechanical forces (i.e., by the inflicted trauma vector) can cause a direct epithelial and endothelial lung cell injury with a rapid release of endogenous danger-associated molecular patterns (DAMPs). In the case of infections, alongside DAMPs, pathogens and their associated molecular patterns (PAMPs) can be released and enter the respiratory system. In the pulmonary micromilieu, a vast amount of different microorganisms meet with ideal “culture” conditions and, therefore, can readily multiply, resulting in a “vicious circle” of the generation of more PAMPs and DAMPs. Both DAMPs and PAMPs will launch an inflammatory pulmonary and systemic response, which in turn can induce further indirect epithelial and endothelial lung damage by an excessive immune response (24, 25). The created pro-inflammatory milieu can have a negative impact on cell adhesion junctions, particularly on VE-cadherin, the components of which will become phosphorylated, finally resulting in “loosening” of the bonds between endothelial cells (26). Cellular damage together with the loss of cellular junctions and disrupted cellular barriers results in increased vascular permeability and enhanced air–blood distances, both of which are key players in ARDS pathogenesis. Increased vascular permeability leads to extravascular (first in the interstitial, then in the alveolar space) accumulation of protein-rich fluid, in this way initiating the exudative phase of ARDS. In addition, the impaired alveolar–capillary barrier facilitates leukocyte and erythrocyte migration into the alveolar space. The edematous fluid leads to hyaline membrane formation and surfactant dysfunction, the latter being associated with an increased risk for atelectasis. Edema, inflammation, and hyaline membranes are hallmarks of diffuse alveolar damage (DAD), which is a pathomorphological correlate of ARDS. Following the exudative phase, hyperplasia of alveolar cells type 2 (AT2) will occur, inducing the proliferative phase of ARDS, which will culminate in interstitial fibrosis and a consequent reduction of the gas-exchange pulmonary area and significant reduction in respiratory function (27).

## Coronavirus Morphopathology

Coronaviruses, members of the *Coronaviridae* family, are enveloped RNA viruses, which are able to cause respiratory infections in humans (28). A natural reservoir of coronaviruses has been proposed in bats (28, 29). Based on the genomic sequence analysis data, the animal-to-human transmission was possible because of the genetic modification of the *Spike* gene (30). The *Spike* gene encodes the Spike protein, which is essential for receptor binding and fusion with the cellular membrane of the target cells. The SARS-CoV and SARS-CoV-2 mechanism of cell entry is based on the angiotensin-converting enzyme 2 (ACE2) receptor interaction with the Spike protein. This interaction allows the SARS-CoV and SARS-CoV-2 to infect mainly the ciliated bronchial epithelial cells and type II pneumocytes (31, 32). By contrast, MERS-CoV uses dipeptidyl peptidase 4 (DPP4, also known as CD26) as a receptor to infect the nonciliated bronchial epithelial cells and type II pneumocytes (33). Despite the fact that many people remain asymptomatic during the infection with SARS-CoV or MERS-CoV, patients with comorbidities, including hypertension, diabetes mellitus, and coronary heart disease (34), as well as males and adults aged over 60 (35, 36) are prone to develop a viral pneumonia associated with a subsequent “cytokine storm.” The latter will surpass the detrimental impact of the virus itself on the patient outcome, thus being associated with a high mortality rate.

### Innate Fluid Phase Response: Complement and Coagulation

#### Trauma-Induced Acute Respiratory Distress Syndrome

The complement system plays an important role in orchestrating the immune response in the lungs. Systemic complement activation has been documented in patients with ARDS and those at risk for ARDS after trauma (37–39). Activation of the complement cascade with the generation of anaphylatoxins as reflected by an enhanced complement factor C3a/C3 ratio in severely traumatized patients was predictive for ARDS development (40). Anaphylatoxins also contribute to increased permeability of the blood–air barrier, with subsequent fluid leakage in the alveolar space, recruitment of immune cells, and stimulation of pro-inflammatory cytokines production (37). An increased level of the complement activation product C5a in bronchoalveolar lavage fluids (BALFs) was observed in patients with traumatic pulmonary contusion. Interestingly, the level of C5a could be correlated with the size of the contused pulmonary area, namely when the injured area exceeded 24% in the CT scan, higher C5a levels could be detected (41).

In a model of trauma-induced HS in nonhuman primates, the PaO_2_ in blood–gas analyses was improved as well as the associated systemic inflammatory cytokine response by blockade of the central complement component C3 (by a compstatin analog), indicating some improvement in the pulmonary blood–gas exchange (42). In murine trauma studies, a significant increase in C5a plasma levels was observed within 24 h after blunt chest trauma. Being a potent chemotactic agent, C5a leads to an elevated and uncontrolled immune cell recruitment in the lung tissue, which correlates with the degree of lung injury. Consequently, it was demonstrated that anti-C5a treatment immediately after blunt chest trauma results in less neutrophil infiltration in the lung tissue, thereby limiting the immune-induced pulmonary injury (41, 43). C5a blockage was also associated with lower interleukin (IL)-6 plasma levels immediately following blunt chest trauma (41).

A hypothesis-free gene expression analysis of lung tissue in rats, conducted 12 h after a blunt chest trauma, revealed a significantly elevated genomic expression of C3, as well as an upregulation of IL-1α, IL-1β, and tumor necrosis factor (TNF)-α receptor (44). Further studies demonstrated elevated C3a, C3a receptor, and HMGB1 levels in the lungs post trauma and that complement blockade could reduce the HMGB1 expression and translocation in the trauma-injured lungs (45).

The role of complement in maintaining the blood–air barrier was also investigated in ischemia/reperfusion lung injury in rodents. It was demonstrated that systemic complement depletion by cobra venom factor (CVF) decreases the pulmonary permeability and edema, through maintenance of integrity of the tight junctions and reduction of the wet/dry ratio and the total protein content in BALF (46) indicative of an overall improved air–blood barrier.

As an overall limitation, it has to be noted that in retranslational animal modeling of ARDS (47) and, in particular, of trauma-induced ARDS, achieving the Berlin ARDS criteria remains challenging and such models frequently require a second hit injury (such as a pathogen or aggressive ventilation) in addition to a first hit (including trauma and HS) (48). Thus, only limited data of the role of complement in clinically relevant trauma-induced ARDS models are currently available.

#### Coronavirus-Induced Acute Respiratory Distress Syndrome

Recently, the complement system has been shown to also be of importance in SARS-CoV-induced ARDS (24, 49). A seminal murine study on SARS-CoV-infected mice proved the activation of the complement cascade already during the first post-infection day (50). Furthermore, C3 knockout mice displayed significantly less weight loss and less respiratory dysfunction. In comparison with the control mice, the C3 knockout littermates demonstrated less neutrophil and monocyte lung infiltration as well as lower amounts of IL-6, IL-1α, TNF, and granulocyte colony-stimulating factor in lung tissue (G-CSF) (50). Interestingly, both wild-type and C3 knockout mice following infection with SARS-CoV exhibited similarly high expression of macrophage inflammatory protein 1 alpha (MIP1a), MIP1b, and monocyte chemoattractant protein-1 (MCP1), suggesting no involvement of C3 in this particular inflammatory signaling. Of note, C3 absence was reported to have no influence on viral replication (50).

Another study investigated the importance of the C5a-C5a receptor (C5aR) axis in human DPP4 transgenic mice infected with MERS-CoV (51). C5aR blockage resulted in a decrease of macrophage infiltration in the lung tissues and a reduction of the sera levels of IL-1β, TNF, interferon (IFN)-γ, IL-12, and MCP-1. However, C5aR blockade failed to influence the sera levels of IL-6, IL-10, or IP-10. The histopathological analysis revealed some reduced alveolar damage in the C5aR blockade group in comparison with untreated mice. Noteworthy, less viral antigen expression with a lower viral titer in the lung tissue was observed in the treatment group (51).

A very recent study of five patients infected with SARS-CoV-2 reported evidences for complement-mediated microvascular injury in the lung and/or skin tissue, because deposits of C5b–9, C4d, and MASP2 were detected through immunohistological analysis (52). Furthermore, the authors described the histological pattern of the lung injury of the five patients as being a pauci-inflammatory septal capillary injury with an abundant fibrin septal capillary deposition together with neutrophil infiltration in the interalveolar septal space. Moreover, it is likely that complement-mediated microangiopathy might be involved in the exaggerated activation of the coagulation cascade. This could be one of the reasons why, in critically ill SARS-CoV-2 patients, increased D-dimer levels were reported (53–55).

#### Coagulation Cascade

It is tempting to speculate that the interplay between the complement and coagulation systems (56) plays a distinct role in ARDS pathology. Firstly, thrombin is involved in C5a generation in a C3-independent manner (56), thereby enhancing the immune response. Secondly, the resulting immune response will induce some coagulopathic conditions in the lungs, in which, for example, the urokinase pathway has been proposed to play a pivotal role not only in fibrin turnover but also in the regulation of vascular permeability (57). Examining the gene expression patterns of rat lungs after blunt chest trauma, an augmented expression of the tissue-type plasminogen activator- and plasminogen activator urokinase receptor-encoding genes was observed together with enhanced C3 levels (44). Similarly, transcriptomic analysis of lung tissue from SARS-CoV-infected mice revealed an upregulation of the urokinase pathway (58). While decreased urokinase fibrinolytic activity is implicated in hyaline membrane formation and pulmonary fibrosis, serpine (a major protein in the urokinase pathway) knockout mice were more susceptible to SARS-CoV, exhibited a higher risk for developing lung hemorrhage, and displayed a greater weight loss in comparison with wild-type mice (58). This data demonstrates a subtle balance between fibrosis and fibrinolysis and that a controlled immune response could modulate, if not prevent, the extreme consequences of coagulopathy.

### Innate Response of the “First Line of Defense”

#### Neutrophils

In acute lung injury (ALI and ARDS) after trauma, systemic and pulmonary chemotaxins and complement activation products result in upregulation of endothelial adhesion molecules, including e-/p-selectin and vascular cell adhesion molecule 1 (VCAM-1) (59, 60), and subsequent migration of neutrophils into the interstitium and alveolar space. Upon recruitment, these inflammatory cells are activated and, in turn, release inflammatory cytokines and chemokines, which in concert with the lung endothelium and epithelium and alveolar macrophages mount the pulmonary inflammatory response. Neutrophil activation also leads to phagocytosis, degranulation of inflammatory mediators, release of proteases, production of reactive oxygen species (ROS), and generation of neutrophil extracellular traps (NETs) (25). While neutrophils provide an important defensive immune arsenal, exaggerated neutrophil infiltration and activation in lung tissue is generally associated with a poor patient outcome.

##### Trauma-Induced Acute Respiratory Distress Syndrome

In a murine blunt chest trauma model, an increased number of neutrophils was observed in the lungs (43) within hours after trauma. Interestingly, in various trauma settings, neutrophils undergo a unique pre-activation process termed “priming,” where the complement activation product C5a is regarded as one of the priming agents. Primed neutrophils produce and release low amounts of ROS. However, upon *in vitro* exposure to a second stimulus, the primed neutrophils can dramatically increase their ROS production and release, partly through NADPH oxidase (NOX) 2, eventually inducing a severe vascular and lung tissue injury (61, 62).

Recently, a research group proposed the concept of pulmonary neutrophil compartmentalization, using a rat trauma model (63). Immediately following trauma, the authors observed significant differences in the expression profile of neutrophil surface markers, including selectin and integrin, between the neutrophils from the pulmonary parenchyma and those from the bronchoalveolar space. Furthermore, the elevated neutrophil count in the lungs persisted approximately 72 h after the injury, as well as their activation status (63). Similar findings were reported for systemic circulatory neutrophils in patients with isolated blunt chest trauma. Specifically, it was observed that the circulatory neutrophils remained active 24 h after trauma (64). This observation highlights the importance of the particular post-traumatic timeframe, during which appropriate measures could and should be taken to minimize trauma-induced neutrophil “auto-aggressive” tissue injury.

##### Coronavirus-Induced Acute Respiratory Distress Syndrome

Neutrophils appear also to play a crucial role in coronavirus-induced ARDS. Upon stimulation, neutrophils release NETs, which consist of DNA and citrullinated histones, and mount mainly antimicrobial effects. Regardless of their benefits, NETs are found to cause severe tissue injury, coagulopathy, and barrier dysfunction of the lungs in ARDS (65). An investigation on murine VILI revealed remarkable differences between mechanically ventilated and nonventilated mice. The mice ventilated with a high-tidal volume (24 ml/kg, 100 breaths/min, 0 mmHg PEEP), which caused a barotrauma and volutrauma in the lungs, presented higher levels of markers of NET formation [citrullinated histone-3 (Cit-H3), neutrophil elastase (NE), and DNA] in the lung tissue in comparison with the control group. NET formation was also associated with a higher wet/dry ration and with greater concentrations of proteins, IL-6, and TNF in BALF. Yet, the application of DNase shortly after intubation of the mice was able to prevent the NET formation, thus improving the lung compliance (66). Notably, SARS-CoV-2-infected patients were documented to have elevated levels of NET markers [Cit-H3, myeloperoxidase (MPO)-DNA, and cell-free DNA] in serum in comparison with healthy individuals. Furthermore, MPO-DNA correlated with the absolute neutrophil count, whereas Cit-H3 correlated with the platelet count. In contrast to patients who did not require mechanical ventilation, the mechanically ventilated patients had higher levels of cell-free DNA and MPO-DNA, but not Cit-H3 (67).

In addition to mouse studies that revealed an increase of neutrophils in lung tissues following coronavirus infection (50, 51), an investigation conducted on nonhuman primates infected with SARS-CoV described an accumulation of neutrophils in the lungs. Moreover, in this study, a greater number of neutrophils were observed in the aged macaques (10–19 years old) compared with very few in young macaques (3–5 years old) (68). This data validates the disease severity pattern of humans, where older patients are predisposed to develop a more severe course of the disease (69). In addition, post-mortem analysis demonstrated neutrophils in the alveolar space of SARS-succumbed individuals (70).

Several case series reported a correlation among the severity degree of SARS-CoV-2 infection, neutrophil blood count, and lymphocyte blood count (71, 72). Severely ill patients (respiratory rate ≥30 times/min; oxygen saturation ≤93%; PaO_2_/FiO_2_ ≤300 mmHg) displayed a statistically higher neutrophil count and a significantly lower lymphocyte count, in comparison with nonseverely ill patients. Moreover, the neutrophil‐to‐lymphocyte ratio (NLR) is increasingly regarded as a predictive marker for screening of critically ill coronavirus patients (73–75). The usage of the NLR for ARDS severity screening was also previously shown in a retrospective study of pneumonia- and sepsis-induced ARDS, where an augmented NLR was associated with increased mortality in ARDS patients (76). Taken together, systemic neutrophils and their recruitment to the lungs appear to contribute to the SARS-CoV-2 pulmonary damage.

#### Macrophages

Unlike neutrophils, macrophages are part of the normal alveolar structure, being regarded as the primary innate immune sentinels of the lungs. The resident macrophages of the lungs as well as those recruited from the circulation are known to play an essential role in ALI/ARDS initiation, development, and resolution (27). Initially, macrophages are able to sense DAMPs and PAMPs through pattern recognition receptors, including TLRs and NOD-like receptors (NLRs). Recognition of molecular danger together with the pro-inflammatory pulmonary micromilieu after injury/infection induces a M1 (pro-inflammatory) macrophage polarization, that in turn will support the inflammation (25). Macrophages produce mainly pro-inflammatory cytokines and chemokines and contribute to antigen presentation. In the later resolution phase of ARDS, where the pro-inflammatory reactions are ceasing and the anti-inflammatory ones start to prevail, the macrophages shift from the M1 to M2 (anti-inflammatory) phenotype. M2 macrophages are capable of secreting anti-inflammatory cytokines, clearing the debris and eliminating apoptotic cells through efferocytosis, thus balancing the immune response and contributing to lung tissue repair and regeneration. However, in severe cases, the M1 to M2 shift is lacking, and the resulting persistence of the M1 phenotype is believed to be detrimental for the lung tissue (77).

##### Trauma-Induced Acute Respiratory Distress Syndrome

A study conducted on 56 patients with severe trauma (ISS ≥15) highlighted the correlation between BALF levels of IL-8 and the risk for developing ARDS. It was reported that the patients who progressed to ARDS had significantly higher IL-8 levels in BALF immediately after trauma (at a mean time of 95 min after admission), in comparison with severe trauma patients who did no develop ARDS (78). Interestingly, macrophages are one of the most important sources of IL-8, while IL-8 is a potent chemoattractant for neutrophils (27). Further studies on a HS mouse model addressed the interaction between alveolar macrophages and the recruited neutrophils in the lungs. The authors observed that after HS, the alveolar macrophages upregulate NOD2 expression, which partly contributes to the induction of autophagy in macrophages, with consequent suppression of the inflammatory response. However, upon contact with activated neutrophils, the anti-inflammatory effect of autophagy in alveolar macrophages was diminished, thus potentiating the pro-inflammatory immune response in the lungs (79). Nanobiotechnological research, using peptide-coated gold nanoparticles, provided further understanding of macrophage phenotypes in ARDS. In murine ARDS, treatment with anti-inflammatory nanoparticles (inhibitors of TLR signaling in macrophages) resulted in a reduction of neutrophil lung infiltration, lower levels of M1 phenotype-associated cytokines (IL-12p40, IFN-γ), and higher levels of M2-related cytokines (IL-10) in BALF. Furthermore, the anti-inflammatory nanoparticles induced an M2 macrophage polarization in the HS-injured lungs, with a consequent alleviation of lung inflammation (80). Overall, lung macrophages function as major immune regulators in trauma-induced ARDS.

##### Coronavirus-Induced Acute Respiratory Distress Syndrome

Macrophages have also been proposed to play a crucial role in coronavirus infection pathogenesis. Although macrophages do not possess the entry receptor for coronavirus, they can still be infected through the mechanism termed antibody-dependent enhancement (ADE). ADE occurs because of the interaction between the virus–antibody (spike protein–anti-spike antibody) complex with the respective Fc receptor on the targeted cells. This interaction was observed in both SARS and MERS infections (81, 82). The SARS-infected macrophages fail to support viral replication (81) although maintaining their pro-inflammatory activity *in vitro* (83). However, further analyses are required to determine the importance of the ADE coronavirus mechanism of entry in disease pathogenesis as well as in vaccine development.

A delay in IFN-γ signaling followed by monocyte-macrophage accumulation in the lungs is characteristic for a severe course of SARS in mice (84). This data is validated by post-mortem analysis of severely ill SARS patients, where macrophages were found to predominate in the lungs (85). Moreover, the autopsy analysis revealed the presence of multinucleate cells in the lungs from several lineages, including from CD68-positive macrophages (85).

### Response of the Lung Tissue

#### Alveolar Epithelium

The alveolar surface consists mostly of alveolar cell type 1 (AT1) and AT2, forming together with the capillary endothelial cells the blood–gas barrier. Both cells are able to absorb the excess fluid from the alveolar space, mainly through apical sodium active transport (86) and aquaporins, thus reducing the alveolar edema. However, it was documented that ARDS patients display an impaired resolution of lung edema (87).

##### Trauma-Induced Acute Respiratory Distress Syndrome

In the direct aftermath of blunt chest trauma caused by mechanical forces, a direct lung parenchymal injury can occur. Because AT1 constitutes more than 95% of the alveolar surface area, an AT1 cell injury is inevitable in the trauma setting and can be clinically assessed by measuring the soluble receptor for advanced glycation end product (sRAGE) plasma concentration. Therefore, it was reported that severe trauma patients (ISS ≥16) have significantly elevated sRAGE concentrations in the plasma directly after the trauma, in comparison with healthy individuals. The sRAGE levels also correlated with the volume of lung parenchymal injury (88).

AT2 cells produce the surfactant factor and thereby inhibit the collapse of the alveoli. The surfactant production after trauma is altered quantitatively and qualitatively and thereby can overall contribute to ARDS development (89, 90). Furthermore, the surfactant also harbors several antimicrobial factors and opsonins to assist in the innate immune defense against invading pathogens; most of these factors are decreased in trauma settings, which may facilitate pathogen invasion and amplification (91). Taken together, trauma leads directly or indirectly to an impaired pulmonary epithelial barrier, thereby facilitating progression of ARDS pathophysiology.

##### Coronavirus-Induced Acute Respiratory Distress Syndrome

Regarding COVID-19-associated ARDS, AT1 cells have been proposed as an ideal therapeutic target, because their HMGB1-RAGE-mediated inflammatory response (e.g., enhancement of neutrophil-induced injury) can be inhibited by acetylcholine, heparin, statins, or dexmedetomidine (92). However, the complex RAGE signaling is to date not thoroughly investigated in ARDS patients with COVID-19 (93). For AT2 cells, surfactant protein D is considered as a valid biomarker of AT2 injury. Although, in the case of blunt chest trauma, AT2 can be directly or indirectly injured, no differences in surfactant protein D plasma level have been reported in trauma patients with pulmonary contusion versus healthy individuals (88). By contrast, in patients with coronavirus infection, the AT2 cells are directly damaged by the virus, because they represent one important entry gateway (32). In agreement with this, an increase of surfactant protein D was observed in plasma samples from SARS patients (94).

It should be noted that the direct cytotoxic effect of coronavirus on AT2 or direct mechanical insult during the blunt chest trauma on AT2 and AT1 will initiate a local inflammatory response, which, in severe cases, can progress and cause a global epithelial injury in the lungs. The primary consequences of epithelial lung injury are the damage of the blood–gas barrier, increased vascular permeability, and liquid extravasation in the alveolar space.

#### Pulmonary Endothelium

Being part of the blood–gas barrier and providing anatomically and functionally with its integrity, the endothelium is an essential element to be studied in ARDS pathology. Pulmonary endothelium, like alveolar epithelium, can be injured and/or directly activated (e.g., by mechanical forces) or indirectly (e.g., by immune responses).

##### Trauma-Induced Acute Respiratory Distress Syndrome

An important endothelial cell activation marker is angiopoietin 2 (Ang-2), which contributes to an increase in lung vascular permeability. Particularly in the post-traumatic milieu, plasma Ang-2 levels were significantly elevated (95). Moreover, in severely injured patients, Ang-2 together with RAGE plasma concentrations was suggested as predictive markers for ARDS development (95). In patients with higher Ang-2 levels, coagulation abnormalities have been more frequently observed, manifested by a prolonged prothrombin time (PT >15.2 s) and partial thromboplastin time (PTT >36.5 s). Moreover, a positive correlation between Ang-2 and D-dimers was also documented. Taken together, endothelium activation plays an important role in coagulopathy development after trauma (96).

##### Coronavirus-Induced Acute Respiratory Distress Syndrome

The endothelial activation during SARS-CoV-2 infection is currently regarded as a key element in disease pathogenesis, particularly in severe cases (97). Furthermore, the post-mortem analysis of SARS-infected patients revealed clear signs of vascular injury, manifested by endothelial injury and/or denaturation and extravasation of fluids and red blood cells in the alveolar space. In addition, fibrin thrombi have been found in pulmonary vessels, which demonstrate the amplified activation of the coagulation system in endothelial injury settings (98). Similarly, in the post-mortem analysis of SARS-CoV-2-infected patients, histological evidence of generalized endotheliitis was found (99), which might be the underlying reason of thromboembolic events reported in SARS-CoV-2-infected individuals (100). Furthermore and in accordance to the trauma setting, Ang-2 has also been found to be enhanced in COVID-19 patients who required intensive care treatment (101).

## Discussion

**Figure 1:**
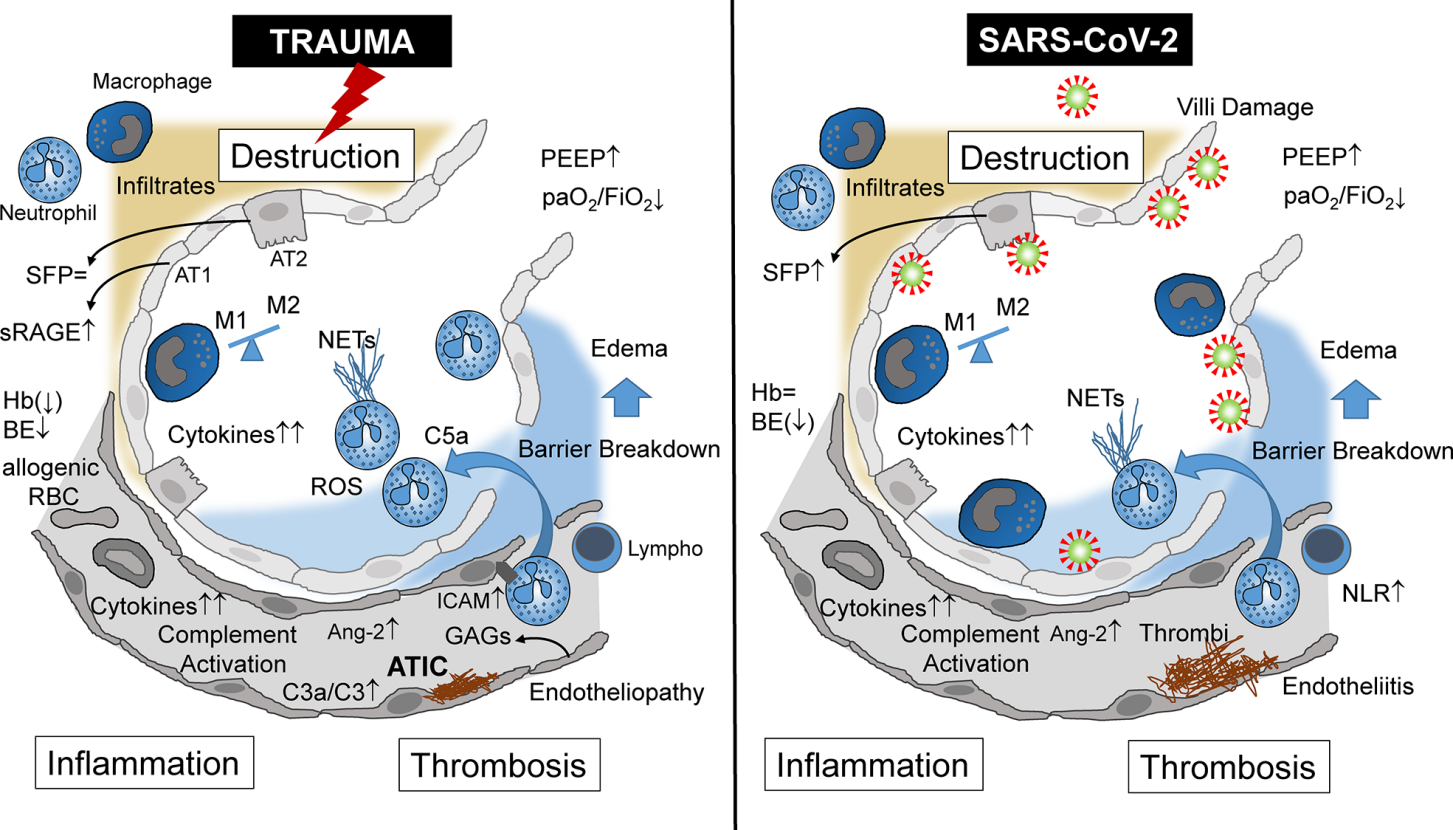
Alveolar immunopathophysiology during ARDS caused by severe tissue trauma (left side) or by SARS-CoV-2 infection (right side) with subsequent destruction, inflammation, thrombosis, and destruction. ARDS, acute respiratory distress syndrome; NETs, neutrophil extracellular traps; NLR, neutrophil/lymphocyte ratio; AT1, alveolar type 1 cells; AT2, alveolar type 2 cells; Ang-2, angiopoietin 2; sRAGE, soluble receptor for advanced glycation end products; SFP, surfactant protein; Hb, hemoglobin; BE, base excess; RBC, red blood cells (transfusion); ATIC, acute trauma-induced coagulopathy; Lymph, lymphocyte; C3, complement component 3; C3a, activated complement 3 (anaphylatoxin); C5a, activated complement 5 (anaphylatoxin); PEEP, positive end-expiratory pressure; paO_2_, partial arterial oxygen pressure; FiO_2_, fraction of inspired oxygen; GAGs, glycosaminoglycans; ICAM, intercellular adhesion molecule.

Focusing on the lungs, ARDS caused by severe tissue trauma shares many immunological features with SARS-CoV-2-induced ARDS (**Table 1**), leading locally and systemically to inflammation, thrombosis, and tissue destruction (**Figure 1**). These three major pathophysiological responses may finally decide whether or not the patient will survive. In Greek mythology, this border between life and death is heavily guarded by the three-headed Cerberus, a vicious dog closely watching the entrance to the underworld. When the three heads of this guard are evident, that is, when inflammation, thrombosis, and destruction are advanced in both the traumatized and viral-infected patient, there is normally the point of no return.

**Table 1:** Similarities and differences of major changes of the innate immune response in clinical and experimental ARDS induced either by severe tissue trauma or SARS-CoV-2 viral infection.

Target organ: lungs	ARDS	
Etiology	Trauma	Coronavirus
Trigger	DAMPs, PAMPs, hypoxia	PAMPs, DAMPs
Onset after insult, days	2–5 (102)	6–12 (71)
Mortality rate, %	21.8 (103)	14.1 (104)
Fluid phase immune response		
- Complement	Complement activation ↑ (37–39) C3a/C3 ratio ↑ (40) C3 inhibition in monkeys: improved BGA values (42)	Complement activation ↑ (24, 49) C3^−/−^ mice: improved lung function (50)
- Coagulation	Activation (thrombosis) (96) DIC (96)	Activation, thrombosis (58) DIC: D-dimers↑ (53–55)
Cellular phase immune response		
- Neutrophil	↑↑ intrapulmonary recruitment and activation (43) NETs ↑ (25) NLR (↑) (105)	↑ intrapulmonary recruitment and activation (50, 51) NETs ↑↑ (67) NLR ↑ (73−75)
- Macrophage	↑ (79) M1 polarization (80) “Cytokine storm” (e.g., IL-8) (78)	↑↑ (81, 82, 85) M1 polarization (84) “Cytokine storm” (84)
Air–blood barrier		
- Lung epithelial cells	AT1 injury: sRAGE ↑ (88) AT2 altered surfactant generation (89, 90)	AT2 entry for SARS-CoV-2 (32) Surfactant protein in plasma ↑ (94)
- Lung endothelial cells	Endotheliopathy ↑ (96) Glycocalyx shedding (96) Ang-2 in plasma ↑ (95)	Endotheliopathy ↑↑ (97) Endotheliitis (99) Ang-2 in plasma ↑ (101)

DAMPs, danger-associated molecular patterns; PAMPs, pathogen-associated molecular patterns; DIC, disseminated intravascular coagulopathy; NETs, neutrophil extracellular traps; NLR, neutrophil/lymphocyte ratio; BGA, blood–gas analysis; AT1, alveolar type 1 cells; AT2, alveolar type 2 cells; Ang-2, angiopoietin 2; sRAGE, soluble receptor for advanced glycation end products.

“ARDS is and is not ARDS”: Besides many similar immunological and pathophysiological characteristics of both etiologies, ARDS induced by SARS-CoV-2 or trauma also exhibits different features (**Table 1**). Whereas traumatic ARDS appears to be neutrophil-driven, at least at the beginning, macrophages as an indirect entry for SARS-CoV-2 appear to be a major inflammatory cell in corona-associated ARDS, and systemic cytokine release is even more pronounced in comparison with the trauma situation.

For future therapeutic strategies against ARDS, it might be important to not only apply all the clinico-scientific knowledge gained in the past for trauma-induced complications but also to transfer the current knowledge increase for COVID-19-associated ARDS to the trauma and other etiologies for the benefit of the patient.

## Author Contributions

The authors have contributed equally.

## Funding

This review has been supported by the DFG collaborative research center CRC1149 project A01 (INST 40/479-2) and Z02 (INST 40/498-2).

## Conflict of Interest

The authors declare that the research was conducted in the absence of any commercial or financial relationships that could be construed as a potential conflict of interest.
